# Acute and post-acute multidisciplinary outcomes of newborns born from mothers with SARS-CoV-2 infection during pregnancy or the perinatal period

**DOI:** 10.1016/j.heliyon.2023.e19206

**Published:** 2023-08-19

**Authors:** Danilo Buonsenso, Giulia Poretti, Francesco Mariani, Arianna Turriziani Colonna, Simonetta Costa, Lucia Giordano, Francesca Priolo, Guido Conti, Angelo Tizio, Daniela Rodolico, Giulia Maria Amorelli, Lorenzo Orazi, Maria Petrianni, Daniela Ricci, Antonio Lanzone, Maurizio Sanguinetti, Paola Cattani, Francesca Raffaelli, Michela Sali, Giuseppe Zampino, Giovanni Vento, Piero Valentini

**Affiliations:** a Department of Woman and Child Health and Public Health, Fondazione Policlinico Universitario A Gemelli IRCCS, Rome, Italy; b Global Health Research Institute, Istituto di Igiene, Università Cattolica Del Sacro Cuore, Rome, Italy; c Dipartimento di Scienze Biotecnologiche di Base, Cliniche Intensivologiche e Perioperatorie, Sezione di Microbiologia, Università Cattolica Del Sacro Cuore, Rome, Italy; d Medicine and Surgery, Università Cattolica Del Sacro Cuore, 00168 Rome, Italy; e Pediatric Resident, Università Cattolica Del Sacro Cuore, 00168 Rome, Italy; f Department of Woman and Child Health and Public Health, Fondazione Policlinico Universitario A Gemelli IRCCS, Rome, Italy; g Institute of Otorhinolaryngology, Università Cattolica Del Sacro Cuore, Fondazione Policlinico “A Gemelli”, IRCCS, Rome, Italy; h Department of Ophthalmology, Gemelli Foundation IRCSS, Catholic University of the Sacred Heart, Rome, Italy; i National Centre of Services and Research for the Prevention of Blindness and Rehabilitation of Low Vision Patients, IAPB Italia Onlus- Fondazione Policlinico Agostino Gemelli IRCCS, Rome, Italy; j Dipartimento di Scienze di Laboratorio e Infettivologiche, Fondazione Policlinico Universitario A Gemelli IRCCS, Rome, Italy

## Abstract

Introduction.

We performed a single-center, prospective, observational study of newborns born from mothers with microbiologically confirmed SARS-CoV-2 infection in pregnancy or at time of delivery to evaluate acute and mid-term multidisciplinary outcomes.

Methods.

Infants were offered a multidisciplinary follow-up consisting of nasopharyngeal Polymerase Chain Reaction test at birth and at 48–72 h of life, auxological and ophthalmological assessments, and serologic testing.

Results.

791 women and their 791 children (52.3% males) were included. Most placentas (94.9%) had abnormal inflammatory findings. 171 (27.3%) and 36 (13.7%) children respectively had pathological TEOAEs in at least one ear and bilaterally, while only four of the 85 children that underwent ABR had pathological findings (4.7%). 64 children underwent fluorescein angiography, which resulted pathological only in 1 case (1.6%). Anti-SARS-CoV-2 IgGs were found in up to 60% of children tested at six months of age. Our findings showed no association between the maternal vaccination status or the presence of maternal symptoms during pregnancy and neonatal outcomes.

Conclusions.

Our study shows that the large majority of newborns exposed to SARS-CoV-2 infection in utero or during the first hours of life have optimal outcomes. Our previous report of abnormal ophthalmologic findings was not confirmed on a larger cohort, while further studies are needed to better characterize audiological outcomes. Further prospective, case-controlled studies are still needed.

## 1 Introduction

Coronavirus disease 2019 (COVID-19) caused by severe acute respiratory syndrome coronavirus 2 (SARS-CoV-2) has been a global health emergency since its declaration as a pandemic on March 11, 2020 by the World Health Organization [1].

Coronaviruses are enveloped, non-segmented, single-stranded ribonucleic acid (RNA) viruses. COVID-19 can affect multiple organs and systems, although it mainly involves the respiratory system, where its involvement can cause a wide range of symptoms from a common cold to severe respiratory distress. Whereas its transmission mainly occurs through human-to-human contact, COVID-19 has shown its potential to transmit via multiple transmission routes [2].

Although SARS-CoV-2 can affect both children and adults, it has been observed that the elderly, men, and individuals with cardiovascular comorbidities are more exposed to the risk of infection, sever illness or death related to COVID-19 [3].

As for other infectious conditions, immune dysregulation might increase the risk of severe illness and death from COVID-19. Notably, pregnant women physiologically acquire changes in their immune system which place them under a condition of immunosuppression. As a result, pregnant women and their infants may be particularly susceptible to SARS-CoV-2 infection and to the development of severe clinical events [4].

Furthermore, the experiences of the two previous notable coronavirus outbreaks, the severe acute respiratory syndrome coronavirus (SARS-CoV) and the Middle East respiratory syndrome coronavirus (MERS-CoV), suggested that pregnant women have an increased risk of adverse outcome, including severe pneumonia, the need for endotracheal intubation, admission to an intensive care unit and death [5].

Since the beginning of COVID-19 pandemic, the association of maternal infection during pregnancy with adverse events and neonatal outcomes has been a major concern. The main symptoms of COVID-19 disease are related to a microcirculatory dysfunction; indeed, infected pregnant women are at increased risk of obstetrical complications, such as spontaneous abortions, premature rupture of membranes, preeclampsia and preterm labor, which appear to be proportional to the severity of the infection. As a consequence, infants born to infected mothers with a more severe clinical course may have a worse outcome [6].

The maternal–fetal interface of SARS-CoV-2 infected women exhibited robust immune responses, which can potentially expose the fetus to a pro-inflammatory background and lead to pathological sequelae, particularly in those organs susceptible to microvascular damages. Neurologic invasion through blood or retrograde neuronal route has been confirmed in the infection of other coronaviruses and the identified receptor for SARS-CoV-2 is present in the nervous system. Moreover, SARS-CoV-2 has a well-established neurotropism that may inflict a wide spectrum of neuropathic effects, potentially including effects on hearing and vision [7].

An increasing number of studies have shown that the COVID- 19 vaccination could effectively prevent severe COVID-19 disease in pregnant women and it has been already proven that antibodies can be transferred across the placenta with a potential protective effect on newborns [8].

In 2020 we preliminary observed the neonatal and mid-term multidisciplinary outcomes of newborns exposed to SARS- CoV-2 infection in utero or during the first days of life [7]. This study aimed to repeat the observation on a larger cohort, comparing neonatal outcomes among infants born to vaccinated and not vaccinated women with COVID-19 infection in pregnancy.

## 2 Methods

### 2.1 Study cohort and data sources

This is a prospective observational cohort study of all children delivered by women with a confirmed diagnosis of SARS-CoV-2 infection assessed at Fondazione Policlinico Universitario A. Gemelli IRCCS of Rome, Italy (FPG) from March 1, 2020 to December 31, 2022. The COVID-19 infection was defined by a positive molecular Polymerase Chain Reaction (PCR) test or an antigen test performed on nasopharyngeal swab during pregnancy or at the time of delivery. FPG is a Regional Referral Center for pregnant women with proven or suspected SARS-CoV-2 infection. The study was approved by the Ethics Committee of the FPG (ID 3104). All patients’ caregivers provided consent to participate to the study.

### 2.2 Inclusion criteria

We included all newborns or fetuses:

– That were delivered from pregnant women with a documented SARS-CoV-2 infection during any period of pregnancy, including time of delivery;
– Whose parents agreed to enroll the children in a multidisciplinary follow-up detailed below
– Whose parents or legal guardians provided written informed consent to participate.

### 2.3 Exclusion criteria

– Newborns delivered from mothers with a suspected but undocumented SARS-CoV-2 infection
– Newborns whose mothers developed symptoms and were diagnosed with SARS-CoV-2 infection after delivery
– Newborns whose parents did not provide written informed consent to participate from a legal guardian.

### 2.4 Interventions

Newborns fulfilling inclusion criteria were entered a short and mid-term follow-up which included:

### 2.5 – neonatal follow-up and pregnancy information

– In order to define if newborns were vertically infected with SARS-CoV-2, in case of mothers infected at the time of delivery, the neonates underwent SARS-CoV-2 real-time polymerase chain reaction on nasopharyngeal swab at birth and between 24 and 48 h of life. Data on pregnancy and delivery were collected from the newborns charts.

### 2.6 – infectious disease follow-up

– We enrolled newborns from mother with proven SARS-CoV-2 infection to a serologic assessment of SARS-CoV-2 specific IgG and IgM/IgA on peripheral blood samples at the age of 3 months or above. We followed the gained experience from other infectious diseases with potential of mother-to-child congenital transmission (e.g. syphilis and toxoplasmosis), in the assumption that we do not know yet the perfect method of confirmation/exclusion of the infection in the newborn/infant. In case of positivity of IgG in the patient's blood, families were invited to repeat IgG dosage every 3 months until 12 months of age to assess if there was clearance of antibodies (meaning maternal IgG rather than self-production). The CE certified version of the Vircell COVID-19 ELISA antibody kit (Vircell Spain S.L.U., Granada, Spain) was used according to manufacturer's recommendation (https://en.virce ll.com/products/covid-19-elisa/), with a cut-off of>1 UI/l for positivity. The kit has a sensitivity of 85% and specificity of 98% for antibody detection.

### 2.7 – audiological follow-up

– The scheduled audiological protocol includes the performance of transient evoked otoacoustic emissions (TEOAEs), a screening test that checks on the functioning of the outer hair cells within the cochlea in response to auditory stimuli. This test is offered at birth or scheduled at a follow-up appointment after discharge in case of logistic inconvenience. Families were also offered to perform the Auditory Brainstem Response (ABR) from the age of 3 months, according to local availability of human and equipment resources, regardless the result of the TEOAEs. We decided to offer this opportunity to be sure to detect late onset sensorineural hearing loss (as documented for CMV congenital infection, also in asymptomatic newborns). Both tests are interpreted according to international guidelines [9], and further details have provided in the Supplementary Material of our previous manuscript [7]. The ABR checks on the function of central nervous system (CNS) auditory pathways. The following auditory thresholds were considered: normal if ≤ 20 dB and hypoacusis (unilateral or bilateral) if ≥ 20 dB (mild 21–40 dB, moderate 41–70 dB, severe 71–90 dB, deep >90 dB). In case of hypoacusis the child was invited to perform a new ABR test at 6 months of life, to confirm or exclude the diagnosis of sensorineural hearing loss and was elected for audiologic care pathway.

### 2.8 – ophthalmologic follow-up

– The ophthalmological assessment of all infants included in the study was conducted in the Pediatric Retina Department of the Catholic University of the Sacred Heart, FPG. All infants born to COVID-19 mothers were offered ophthalmologic examination that consist of fluorescein angiography, computed optical tomography (OCT) and behavioral assessment of visual function. Only infants whose parents gave informed consent for the examination and were cooperative enough to obtain good quality images/functional results, were included in the final analysis. All enrolled infants were checked between 3 and 7 months of age. Parents were educated about the use of oral fuorescein and informed consent was obtained before the procedure. All infants were dilated using tropicamide 1% drops instilled at 10-min intervals approximately 30 min before the imaging session. For those who agreed to the fluorescein angiography (FAG), a 20% fuorescein sodium solution dosage was calculated according to recommendations (7.5 mg/kg under 18 years old) [10], mixed with infant formula milk, and given to the patient 10–20 min before the imaging procedure. Non-contact high-resolution ultra-widefeld scanning laser ophthalmoscope (Optos California, Optos PLC, Dunfermline) was used to obtain fast retinal angiographic images, following application of topical anesthetic drops (oxybuprocaine chlorhydrate 0.4%). The infant was held up to the imaging lens in the “fying baby” position by the examiner supporting the head of the baby with one hand and the other supporting the rest of the body. No lid speculum nor sedation was necessary during the procedure. Medical personnel were always present to monitor the vital signs of the infant. All infants also underwent imaging using the Envisu 2300 portable hand-held SD-OCT (Bioptigen Incorporated, Durham, NC). Scans were obtained following parameters provided by Maldonado et al. [11]. The scan size was 8 × 8 mm and scan density was 1000 A scans/100 B scans. Images were included only if the entire foveal region was visible and image quality was adequate to segment inner (IRL) and outer (ORL) retinal layers.

### 2.9 Statistical analysis

Categorical variables were reported as count and percentage, continuous variables were expressed as mean with standard deviation. For categorical data, statistical associations between them were obtained by Chi-squared tests or Fisher's exact tests. Statistical analysis was performed using IBM SPSS Statistics 25.0 software (IBM Corporation, Armonk, NY, USA). Data have been presented accordind to the Enhancing the QUAlity and Transparency Of health Research (EQUATOR) network guidelines, STROBE checklist for cohort studies (supplementary material).

## 3 Results

### 3.1 Study population

**Fig. 1:**
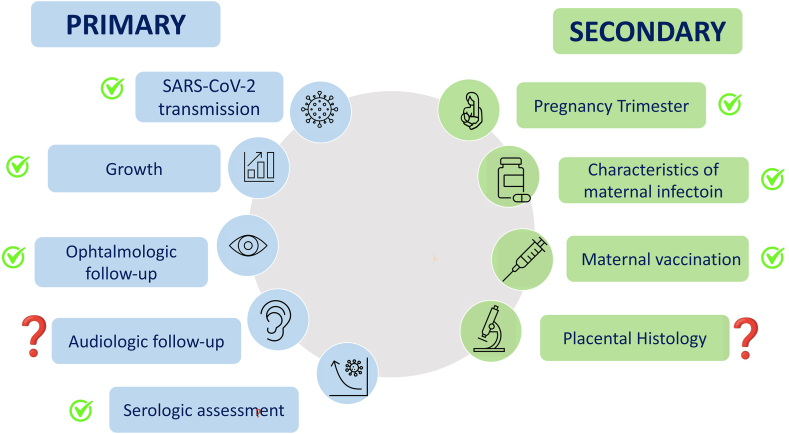
Summary of main findings. Question marks represent partially negative findings that deserve further investigation.

791 women and their 791 children (52.3% males) were included in the study (main results summarized in Fig. 1). The mean maternal age was 33.4 ( ± 5.4) years. The mean gestational age was 38.9 ( ± 1.7) weeks and the prevalence of preterm delivery was 7.3% (58 children). Among the 791 women, 37 (5.0%), 90 (12.2%) and 610 (82.8%) were respectively infected in the first, second and third trimester of pregnancy. 323 (48.9%) women resulted positive to SARS-CoV-2 infection at the time of discharge. 312 (73.2%) women were not vaccinated, therefore 114 (26.8%) received at least 1 dose. Only 8 (1.9%) and 14 (3.4%) newborns tested positive for SARS-CoV-2 PCR at birth and between 24 and 48 h of life. The mean neonatal length, weight and head circumference at birth are reported in Table 1.

**Table 1:** Demographics of study population.

	Mother	Newborn
Mean age (years ± SD)	33.4 ( ± 5.4)	
Genderrowhead		
M		414 (52.3%)
F		377 (47.7%)
Trimester of infection		
I	37 (5.0%)*	
II	90 (12.2%)*	
III	610 (82.8%)*	
	*missing data: 54	
Positive swab at the time of discharge		
	323 (48.9%)*	I swab: 8 (1.9%)* II swab: 14 (3.4%)**
	* missing data: 130	* missing data: 372 ** missing data: 382
Feeding		
Breast Milk		109 (50.9%)*
Artificial milk		36 (16.8%)*
Mixed Milk		69 (32.2%)*
		* missing data: 577
Vaccination		
No	312 (73.2%)*	
yes	114 (26.8%)*	
	* missing data: 365	
Mean gestational age (weeks ± SD)		38.9 ( ± 1.7)
Mean birth weight (g ± SD)		3181.3 ( ± 502.2)
Mean birth head circumference (cm ± SD)		34.2 ( ± 1.4)
Mean birth length (cm ± SD)		49.4 ( ± 2.1)
Prematurity		
No		733 (92.7%)
yes		58 (7.3%)
Birth weight percentile<10°		
No		715 (92.3%)*
Yes		60 (7.7%)*
		* missing data: 16

108 (16.3%) women had fever during the acute phase of the infection, with an average maximum temperature of 37.9 ( ± 0.5) °C. 45 (6.8%) and 41 (6.2%) women respectively experienced anosmia and dysgeusia. Cough affected 80 (12.2%) cases, sore throat 30 (4.6%), rhinitis 55 (8.3%), dyspnea 26 (4.0%) and gastrointestinal symptoms 13 (2.0%) cases. 31 (21.4 %) women, among 145 (18.3%) who underwent chest x-rays, resulted positive at radiological assessment. A small group of women underwent chest ultrasound and CT, as shown in Table 2. Only 2 women (0.3%) were treated with Remdesivir. 23 (2.9%) women received low flow oxygen therapy, 1(0.1%) and 2 (0.3%) women respectively underwent CPAP and intubation. Only 6 (0.8%) women needed Intensive Care unit. The mean value of maternal PCR was 50.6 ( ± 56.7) and the mean value of D-dimer was 2903.6 ( ± 2723. 7). Those characteristics are reported in Table 2.

**Table 2:** Clinical characteristics of maternal SARS-Cov2 infection.

Fever	No	554 (83.7%)*
	yes	108 (16.3%)*
	Max T°C ( ± SD)	37.9 ( ± 0.47)**
		* missing data: 129
		** missing data: 740
Anosmia	No	614 (93.2%)*
	yes	45 (6.8%)*
		* missing data: 132
Dysgeusia	No	618 (93.8%)*
	yes	41 (6.2%)*
		* missing data: 132
Sore Throat	No	629 (95.4%)*
	yes	30 (4.6%)*
		* missing data: 132
Rhinitis	No	604 (91.7%)*
	yes	55 (8.3%)*
		* missing data: 132
Cough	No	576 (87.8%)*
	yes	80 (12.2%)*
		* missing data: 135
Dyspnea	No	632 (96.0%)*
	yes	26 (4.0%)*
		* missing data: 133
Gastrointestinal Symptoms	No	647 (98.0%)*
	yes	13 (2.0%)*
		* missing data: 131
Chest X-Ray performed	No	646 (81.7%)
	yes	145 (18.3%)
Chest X-Ray	Negative	114 (78.6%)*
	Positive	31 (21.4%)*
		* missing data: 646
Chest Ultrasound performed	No	779 (98.5%)
	yes	12 (1.5%)
Chest Ultrasound	Negative	4 (33.3%)*
	Positive	8 (66.7%)*
		* missing data: 779
Chest CT performed	No yes	784 (99.1%) 7 (0.9%)
Chest CT	Negative	2 (28.6%)*
	Positive	5 (71.4%)*
		* missing data: 784
Remdesivir	No	789 (99.7%)
	yes	2 (0.3%)
Low Flow Oxygen	No	768 (97.1%)
	yes	23 (2.9%)
CPAP	No	789 (99.7%)
	yes	2 (0.3%)
Intubation	No	790 (99.9%)
	yes	1 (0.1%)
Intensive Care Unit Admission	No	785 (99.2%)
	yes	6 (0.8%)
Mean CRP value ( ± SD)		50,58 ( ± 56.7)*
		* missing data: 687
Mean D-dimer value ( ± SD)		2903,62 ( ± 2723.7)*
		* missing data: 606

### 3.2 Placenta evaluations

313 women (39.6%) underwent histological examination of the placenta: perivillous fibrin deposition was present in 95 (30.4%) cases, villous hypoplasia in 85 (27.2%), increase in syncytial nodes in 297 (94.9%), intervillous thrombosis in 40 (12.8%) and inflammatory placental lesions in 28 (8.9%) cases, as reported in Table 3.

**Table 3:** Placenta histological characteristics.

Placentar istological examination		
	Not performed	478 (60.4%)
	performed	313 (39.6%)
Perivillous fibrin deposition		
	No	218 (69.6%)
	yes	95 (30.4%)
Villous hypoplasia		
	No	228 (72.8%)
	yes	85 (27.2%)
Increase in 21yncytial nodes		
	No	16 (5.1%)
	yes	297 (94.9%)
Intervillous thrombosis		
	No	273 (87.2%)
	yes	40 (12.8%)
Inflammatory placental lesions		
	No	285 (91.1%)
	yes	28 (8.9%)

### 3.3 Multi-specialistic assessments

For 626 children (89.8%) TEOAEs results were available. Right ear resulted pathological in 133 (21.2%) and left ear in 127 (20.4%) cases. 171 (27.3%) and 36 (13.7%) children respectively had pathological TEOAEs in at least one ear and bilaterally.

85children underwent ABR which resulted pathological in 4 cases (4.7%). 64 children underwent fluorescein angiography, which resulted pathological only in 1 case (1.6%). The ophthalmological and audiological follow-up characteristics are reported in Table 5.

**Table 4:** Infant's sierology results.

Serology at 1 month		
	Not performed	778 (98.4%)
	performed	13 (1.6%)
	Negative	11 (84.6%)
	Positive	2 (15.4%)
Serology at 3 months		
	Not performed	695 (87.9%)
	performed	96 (12.1%)
	Negative	65 (67.7%)
	Positive	31 (32.3%)
Serology at 6 months		
	Not performed	765 (96.7%)
	performed	26 (3.3%)
	Negative	10 (3.5%)
	Positive	16 (61.5%)

**Table 5:** Neonatal result of ABR and FAG.

FAG		
	Not performed	727 (91.9%)
	performed	64 (8.1%)
	Negative	63 (98.4%)
	Positive	1 (1.6%)
ABR		
	Not performed	706 (89.3%)
	performed	85 (10.7%)
	Negative	81 (95.3%)
	Positive	4 (4.7%)

13 (1.6%), 96 (12.1%) and 26 (3.3%) children respectively underwent serological examination at the first, third and sixth month after birth. Serological examination resulted positive in 2 (15.4%) children at the first month, 31 (32.3%) at the third month and 16 (61.5%) at the sixth month as shown in Table 4.

A comparison between newborn's outcome and placenta histological characteristics and the different trimester of maternal infection is reported in Table 6. The only statistically significative association observed was the one related to the pathological ABR result and the second trimester of infection (p = 0.04).

**Table 6:** Association between newborn's outcome and placenta histological characteristics and the different trimester of maternal infection.

	I Trimester (N = TOT)	II Trimester	III Trimester	p-value
**Pathological TEOAEs at least 1 ear**	6 (21.4%)	19 (22.6%)	138 (29.1%)	0.36
**Bilateral pathological TEOAEs**	2 (7.1%)	9 (10.7%)	73 (15.4%)	0.29
**Birth weight percentile** **< 10°**	5 (13.5%)	7 (7.9%)	41 (6.9%)	0.31
**Perivillous fibrin deposition**	3 (42.9%)	3 (20.0%)	83 (30.3%)	0.53
**Villous hypoplasia**	4 (57.1%)	3 (20.0%)	74 (27.0%)	0.17
**Increase in yncytial nodes**	7 (100.0%)	13 (86.7%)	263 (96.0%)	0.19
**Intervillous thrombosis**	1 (14.3%)	2 (13.3%)	33 (12.0%)	0.98
**Inflammatory placentar lesions**	1 (14.3%)	3 (20.0%)	23 (8.4%)	0.28
**Prematurity**	2 (5.4%)	6 (6.7%)	46 (7.5%)	0.86
**Neonatal abnormal FAG**	0 (0.0%)	0 (0.0%)	1 (1.9%)	0.90
**Neonatal abnormal ABR**^a^	0 (0.0%)	2 (16.7%)	1 (1.5%)	0.04

a for one mother of children with pathological ABR, trimester of Infection was unavailable.

A comparison between neonatal outcomes in patients born from mother that were affected by a symptomatic infection and mother affected by an asymptomatic infection is reported in Table 7. Another comparison between neonatal outcomes of children born from vaccinated and unvaccinated women is reported in Table 8. Our findings showed no association between the maternal vaccination status or the presence of maternal symptoms during pregnancy and neonatal outcomes.

**Table 7:** Association between newborn's outcome and placenta histological characteristics and the maternal vaccination status.

	Vaccinated mother	Not vaccinated mother	p-value
**Pathological TEOAEs in at least 1 ear**	45 (211%)	32 (30.8%)	0.06
**Bilateral pathological TEOAEs**	22 (103%)	18 (17.3%)	0.08
**Birth weight percentile** **< 10°**	28 (9.2%)	6 (5.4%)	0.22
**Perivillous fibrin deposition**	64 (34.8%)	10 (30.3%)	0.62
**Villous hypoplasia**	51 (27.7%)	7 (21.2%)	0.44
**Increase in yncytial nodes**	178 (96.7%)	31 (93.9%)	0.35
**Intervillous thrombosis**	27 (14.7%)	2 (6.1%)	0.27
**Inflammatory placental lesions**	18 (9.8%)	4 (12.1%)	0.75
**Prematurity**	16 (5.1%)	9 (7.9%)	0.28
**Neonatal abnormal FAG**	1 (2.2%)	0 (0.0%)	1.00
**Neonatal abnormal ABR**	3 (4.8%)	0 (0.0%)	1.00

*for one mother of children with pathological ABR, vaccination status was unavailable.

**Table 8:** Association between newborn's outcome and placenta histological characteristics and maternal presence of symptoms at time of delivery.

	Mother with no symptoms at time of delivery	Mother with symptoms at time of delivery	p-value
**Pathological TEOAEs in at least 1 ear**	150 (27.6%)	17 (29.3%)	0.78
**Bilateral pathological TEOAEs**	73 (13.4%)	10 (17.2%)	0.43
**Birth weight percentile** **< 10°**	51 (8.1%)	3 (3.4%)	0.12
**Perivillous fibrin deposition**	66 (28.1%)	17 (29.8%)	0.80
**Villous hypoplasia**	63 (26.8%)	18 (31.6%)	0.47
**Increase in yncytial nodes**	222 (94.5%)	55(96.5%)	0.74
**Intervillous thrombosis**	29 (12.3%)	9 (15.8%)	0.49
**Inflammatory placental lesions**	20 (8.5%)	6 (10.5%)	0.63
**Prematurity**	46 (7.1%)	8 (9.0%)	0.53
**Neonatal abnormal FAG**	0 (0.0%)	0 (0.0%)	
**Neonatal abnormal ABR**	4 (6.1%)	0 (0.0%)	0.58

## 4 Discussion

In this study, we showed results from a comprehensive multidisciplinary follow-up of a large cohort of newborns born from mothers with SARS-CoV-2 infection during different pregnancy trimester or in the immediate peripartum. Overall, we found optimal neonatal outcomes without spotting any significant unusual pattern of abnormalities associated with SARS-CoV-2 in pregnancy. These data reflect the impact of early exposure of newborns in utero or soon after birth and may serve as comparison for newborns never exposed in utero and infected in the late neonatal period.

Vertical transmission of SARS-CoV-2 can occur in utero before the onset of labor (intrauterine) or during labor or delivery (intrapartum). Horizontal transmission occurs after the first 48 of life either through contact with the mother or other people (postpartum). Although some cases have been documented in literature, intrauterine transmission still appears to be rare, and most newborns become infected after birth when in contact with infected caregivers [12]. For intrauterine transmission of a pathogen to occur, it is necessary to reach and pass the placenta. The higher the quantity of replicating virus is in the bloodstream; the higher will be the chance of transmission. There are two factors protecting the fetus from the transmission: SARS-CoV-2 is not generally associated with high levels of viremia and, moreover, it is possible that the placenta does not express high levels of primary factors facilitating entry of the coronavirus into cells, such as ACE2 and TMPRSS2 [13]. Of note, our cohort confirm, as did our previous findings [14] that vertical transmission is possible, although rare, as some children had positive SARS-CoV-2 swabs within the first 24 h of life and/or before discharge (between 24 and 48 h of life). However, in this cohort no severe cases with symptoms associated to the infection were noted.

Although vertical intrauterine transmission of SARS-CoV-2 seems rare, since the beginning of the pandemic there has always been concern on the possible neonatal outcome. So far, an increased risk of preeclampsia, preterm delivery and stillbirth have been described among infected pregnant women compared with the rest of the pregnant population [15]. Supporting the role of SARS-CoV-2 infection as causative agent of the adverse outcomes, significant histological alterations, such as necrosis, intervillous inflammation and fibrin deposition, were observed in placentas from pregnant women infected with SARS-CoV-2 who experienced the complications described above [16].

At our institution we also analyzed placentas delivered by infected mothers [7]: we did not find an association between recurrent placental histological alterations and neonatal adverse outcomes. During the last two years we augmented the strength of our findings by expanding the sample: although changes in placental histology (deposition of perivillous fibrin, villous hypoplasia, enlarged syncytial nodes, thrombosis and intervillous inflammatory lesions) were found in a large group of mothers, no significant association was found between these and adverse neonatal outcomes. Besides, no stillbirth was recorded and nothing of these complications was recorded as statistically significant and influencing on the baby's health.

Giuliani et al. analyzed the outcomes of a multinational cohort and found that women with COVID-19 diagnosis had a higher rate of cesarean delivery and pregnancy-related complications such as preterm birth, than women without COVID-19 diagnosis [17]. Again, Norman et al. [18], after matching infants by maternal characteristics, found that some neonatal outcomes such as assisted ventilation at birth and respiratory distress syndrome were significantly more common in infants of SARS-CoV-2–positive women than in infants of comparator women, although preterm delivery induced by maternal COVID-19 was identified to mediate 89.3% of the estimated association between maternal infection and neonatal respiratory outcome.

As regards neonatal neurological outcomes, there are controversial data in literature. Some case reports document severe neonatal neurological outcomes associated with maternal SARS-CoV-2 infection, with demonstration of placental damage, maternal inflammatory response or presence of the virus in the neonatal CNS [18]. In a large cohort of 222 offspring of SARS-CoV-2 infected mothers, compared with the offspring of 7550 of non-infected mothers, neurodevelopmental disorders diagnosis at 12 months of life has been found significantly more common among exposed offspring, even after adjustment for preterm birth [19]. In fact, the developing CNS seems particularly sensitive to SARS-CoV-2 infection, especially if the infection occurs in the first trimesters, due to the presence of high ACE2 expression at this level. The interaction between the virus and the cerebral ACE2 in a critical time window for fetal brain development could alter the integrity of the blood brain barrier leading to cerebral hemorrhages, impaired neurological development, causing early-onset seizures, acquired microcephaly, sudden infant death [20].

With this evidence in the background, we conducted this study to explore the impact of COVID-19 infection in pregnancy on neonatal and infants' outcomes.

In our first cohort, the preliminary findings [7] showed an association between maternal SARS-CoV-2 infection and an increased risk of pathological ophthalmologic assessment at 6 months. In this study, as the sample was enlarged, this association was disconfirmed. On the other hand, once enlarged the sample of infants submitted to audiologic assessment, a statistically significant association was found between the trimester of maternal SARS-CoV-2 infection and the risk of a pathologic ABR result at 6 months. ABR is widely felt to be an objective technique for predicting hearing loss and, according to the Joint Committee on Infant Hearing Position Statement 2019, infant who do not pass in one or both ears on the rescreen, immediate referral to a pediatric audiologist with capabilities for a diagnostic ABR testing should be made for the hearing impairment diagnosis [21]. We found pathological ABRs at 6 months of life in 4.7% of 85 newborns exposed in utero to SARS-CoV-2 infection who had undergone audiological examination; this percentage would indicate an audiological deficit much higher than that reported in the general population equal to about 0.1% [22]. Our findings suggest that more data are necessary to rule out whether COVID-19 infection in pregnancy is a risk factor for hypoacusis in children.

Recently, Firestein et al. [23] analyzed a cohort of 407 infants born to 403 mothers and no association was found between mild or asymptomatic maternal SARS-CoV-2 infection during pregnancy and infant parent-reported neurodevelopment. Although a more detailed neurological examination performed by a specialized neuropsychiatrist is still ongoing, when our 791 children were assessed by a general pediatrician during our follow-up, no neurocognitive major problems have been documented.

Our study also confirmed that infants born to mothers with SARS-CoV-2 infection in pregnancy in most cases, although not always, have persisting IgG antibodies at six months of follow-up even when they had no other known infections, confirming that these antibodies can protect the placenta and somehow protect the child during the first months of life [24].

Overall, our study has practical implications. Overall, the mostly positive outcomes from our cohort would suggest that clinicians involved in the follow-up of children born from mothers with COVID-19 in pregnancy would mostly focus their efforts on the still unanswered questions. Studies should focus on the auditory outcomes, and on neurocognitive development, which in our paper has been only partially assessed. Secondly, these data maybe important to provide prognostic information to mothers with COVID-19 during pregnancy, supporting attention but overall reassuring the parents. Third, while the public may receive the results of this study as encouraging, it remains still important to highlight that some outcomes need more study and preventing SARS-CoV-2 infection during pregnancy remains a priority. In this regard, counselling of pregnant women or those planning a pregnancy remains a public health priority, as studies have demonstrated that even for historical infections with severel neonatal outcomes are poorly recognized by women [25].

In fact, negative outcomes from COVID-19 in pregnancy have been documented by other cohorts. A secondary analysis of a multinational, cohort study on all consecutive pregnant women with laboratory-confirmed COVID-19 from February 1, 2020 to April 30, 2020 from 73 centers from 22 different countries, found that early gestational age at infection, maternal ventilatory supports and low birthweight are the main determinants of adverse perinatal outcomes in fetuses with maternal COVID-19 infection, while the risk of vertical transmission was negligible as in our cohort [26]. Another retrospective cohort of 61 pregnant women positive for SARS-CoV-2 infection at the time of delivery, found that symptomatic pregnant women with SARS-CoV-2 infection at the delivery have a slightly higher but non-significant rate of preterm delivery, cesarean section, as well as lower neonatal birth weight and Apgar score, compared with asymptomatic women [27].

Our study has limitations to address: the most important one is that only a limited number of children completed the audiologic and ophthalmologic assessments, due to the limited availability of spots to perform the exams and the low compliance of the parents of children who appeared completely well-being. Besides, not all the examinations were performed at the same age. Last, we did not include a control group of women that never had COVID-19 during pregnancy. However, credit goes to our study group to be the first to perform such a complex multidisciplinary follow-up and to provide data in this field also useful to child neurologists, ophthalmologists, othorinolaringoiatric specialists, not just to pediatricians and parents.

## 5 Conclusion

In conclusion, our study showed that the neonatal and mid-term multidisciplinary outcomes of newborns exposed to SARS-CoV-2 infection in utero or during the first hours of life are mostly positive, except for audiological findings which were abnormal in about 4,7% of cases. Since our findings are preliminary, further prospective, longitudinal, case-controlled studies of cohorts followed-up for years are necessary to better understand the clinical outcomes of children exposed to SARS-CoV-2 in utero and in the early postnatal life and to clarify the controversies of literature.

The study has been approved by the ethic committee of Gemelli University Hospital of Rome, Italy (ID 3104).

## Author. Contribution statement

Danilo Buonsenso; Piero Valentini: Conceived and designed the experiments; Wrote the paper.

Francesco Mariani: Performed the experiments; Analyzed and interpreted the data.

Maurizio Sanguinetti: Performed the experiments; Contributed reagents, materials, analysis tools or data.

Michela Sali: Conceived and designed the experiments; Performed the experiments; Contributed reagents, materials, analysis tools or data.

Giulia Poretti; Arianna Turriziani Colonna; Simonetta Costa; Lucia Giordano; Francesca Priolo; Guido Conti; Angelo Tizio; Daniela Rodolico; Giulia Maria Amorelli; Lorenzo Orazi; Maria Petrianni; Daniela Ricci; Antonio Lanzone; Paola Cattani; Francesca Raffaelli; Giuseppe Zampino; Giovanni Vento: Performed the experiments.

## Data availability statement

Data will be made available on request.

## Declaration of competing interest

The authors declare that they have no known competing financial interests or personal relationships that could have appeared to influence the work reported in this paper.

## References

[1] (2020). WHO Director-General’s opening remarks at the media briefing on COVID-19.

[2] Mirbeyk M., Saghazadeh A., Rezaei N. (2021). A systematic review of pregnant women with COVID-19 and their neonates. Arch Gynecol Obstet. luglio.

[3] Kumar D., Verma S., Mysorekar I.U. (2023). COVID-19 and pregnancy: clinical outcomes; mechanisms, and vaccine efficacy. Transl Res. gennaio.

[4] Wang C.L., Liu Y.Y., Wu C.H., Wang C.Y., Wang C.H., Long C.Y. (2021). Impact of COVID-19 on pregnancy. Int. J. Med. Sci..

[5] Juan J., Gil M.M., Rong Z., Zhang Y., Yang H., Poon L.C. (2020). Effect of coronavirus disease 2019 (COVID-19) on maternal, perinatal and neonatal outcome: systematic review. Ultrasound Obstet Gynecol. luglio.

[6] De Rose D.U., Salvatori G., Dotta A., Auriti C. (2022). SARS-CoV-2 Vaccines during pregnancy and breastfeeding: a systematic review of maternal and neonatal outcomes. Viruses. 5 marzo.

[7] Buonsenso D., Costa S., Giordano L., Priolo F., Colonna A.T., Morini S. (2022). Short- and mid-term multidisciplinary outcomes of newborns exposed to SARS-CoV-2 in utero or during the perinatal period: preliminary findings. Eur J Pediatr. aprile.

[8] Zhang D., Huang T., Chen Z., Zhang L., Gao Q., Liu G. (2023). Systematic review and meta-analysis of neonatal outcomes of COVID-19 vaccination in pregnancy. Pediatr Res. 3 gennaio.

[9] From laboratory to clinic: a large scale study of distortion product otoacoustic emissions in ears with normal hearing and ears with hearing loss - PubMed [Internet]. [citato 8 maggio 2023]. Disponibile su: https://pubmed.ncbi.nlm.nih.gov/9416447/. 10.1097/00003446-199712000-00003 9416447

[10] Marmoy O.R., Henderson R.H., Ooi K. (2022). Recommended protocol for performing oral fundus fluorescein angiography (FFA) in children. Eye (Lond). gennaio.

[11] Maldonado R.S., Izatt J.A., Sarin N., Wallace D.K., Freedman S., cotton C.M. (2010). Optimizing hand-held spectral domain optical coherence tomography imaging for neonates, infants, and children. Invest Ophthalmol Vis Sci. maggio.

[12] Jamieson D.J., Rasmussen S.A. (2022). An update on COVID-19 and pregnancy. Am J Obstet Gynecol. febbraio.

[13] Term Human Placental Trophoblasts Express SARS-CoV-2 Entry Factors ACE2, TMPRSS2, and Furin | mSphere [Internet]. [citato 24 aprile 2023]. Disponibile su: https://journals.asm.org/doi/full/10.1128/mSphere.00250-21. 10.1128/mSphere.00250-21 PMC8546705 33853873

[14] Costa S., Giordano L., Bottoni A., Tiberi E., Fattore S., Pastorino R., Simone N.D., Lanzone A., Buonsenso D., Valentini P., Cattani P., Santangelo R., Sanguinetti M., Scambia G., Vento G. (2022 May 17). Vertical transmission of SARS-CoV-2 during pregnancy: a prospective Italian cohort study. Am. J. Perinatol..

[15] Wei S.Q., Bilodeau-Bertrand M., Liu S., Auger N. (2021). The impact of COVID-19 on pregnancy outcomes: a systematic review and meta-analysis. CMAJ. 19 aprile.

[16] SARS-CoV-2 placentitis, stillbirth, and maternal COVID-19 vaccination: clinical–pathologic correlations - American Journal of Obstetrics & Gynecology [Internet]. [citato 24 aprile 2023]. Disponibile su: https://www.ajog.org/article/S0002-9378(22)00800-6/fulltext. 10.1016/j.ajog.2022.10.001 PMC9554221 36243041

[17] Effects of prenatal exposure to maternal COVID-19 and perinatal care on neonatal outcome: results from the INTERCOVID Multinational Cohort Study — Keio University [Internet]. [citato 8 maggio 2023]. Disponibile su: https://keio.pure.elsevier.com/en/publications/effects-of-prenatal-exposure-to-maternal-covid-19-and-perinatal-c. 10.1016/j.ajog.2022.04.019 PMC9017081 35452653

[18] Norman M., Navér L., Söderling J., Ahlberg M., Hervius Askling H., Aronsson B. (25 maggio 2021). Association of maternal SARS-CoV-2 infection in pregnancy with neonatal outcomes. JAMA.

[19] Edlow A.G., Castro V.M., Shook L.L., Kaimal A.J., Perlis R.H. (2022 Jun 1). Neurodevelopmental outcomes at 1 Year in infants of mothers who tested positive for SARS-CoV-2 during pregnancy. JAMA Netw. Open.

[20] Benny M, Bandstra ES, Saad AG, Lopez-Alberola R, Saigal G, Paidas MJ, et al. Maternal SARS-CoV-2, placental changes and brain injury in 2 neonates. Pediatrics. 6 aprile 2023;e2022058271. . 10.1542/peds.2022-058271 PMC10467358 37021494

[21] Year 2019 Position Statement: Principles and Guidelines for Early Hearing Detection and Intervention Programs. The Joint Committee on Infant Hearing ; 10.15142/fptk-b748. 10943021

[22] Shave S., Botti C., Kwong K. (2022 Apr). Congenital sensorineural hearing loss. Pediatr Clin North Am.

[23] Firestein M.R., Shuffrey L.C., Hu Y., Kyle M., Hussain M., Bianco C. (2023). Assessment of neurodevelopment in infants with and without exposure to asymptomatic or mild maternal SARS-CoV-2 infection during pregnancy. JAMA Netw Open. 1 aprile.

[24] Nielsen S.Y., Petersen L.H., Murra M., Hvidman L., Helmig R.B., Møller J.K. (2023). Transplacental transfer of SARS-CoV-2 antibodies: a cohort study. Eur J Clin Microbiol Infect Dis. marzo.

[25] Mazzitelli M., Micieli M., Votino C., Visconti F., Quaresima P., Strazzulla A., Torti C., Zullo F. (2017). Knowledge of human cytomegalovirus infection and prevention in pregnant women: a baseline, operational survey. Infect. Dis. Obstet. Gynecol..

[26] Di Mascio D., Sen C., Saccone G., Galindo A., Grünebaum A., Yoshimatsu J., Stanojevic M., Kurjak A., Chervenak F., Rodríguez Suárez M.J., Gambacorti-Passerini Z.M., Baz M.L.A.A., Aguilar Galán E.V., López Y.C., De León Luis J.A., Hernández I.C., Herraiz I., Villalain C., Venturella R., Rizzo G., Mappa I., Gerosolima G., Hellmeyer L., Königbauer J., Ameli G., Frusca T., Volpe N., Luca Schera G.B., Fieni S., Esposito E., Simonazzi G., Di Donna G., Youssef A., Della Gatta A.N., Di Donna M.C., Chiantera V., Buono N., Sozzi G., Greco P., Morano D., Bianchi B., Lombana Marino M.G., Laraud F., Ramone A., Cagnacci A., Barra F., Gustavino C., Ferrero S., Ghezzi F., Cromi A., Laganà A.S., Laurita Longo V., Stollagli F., Sirico A., Lanzone A., Driul L., Cecchini D.F., Xodo S., Rodriguez B., Mercado-Olivares F., Elkafrawi D., Sisti G., Esposito R., Coviello A., Cerbone M., Morlando M., Schiattarella A., Colacurci N., De Franciscis P., Cataneo I., Lenzi M., Sandri F., Buscemi R., Gattei G., Sala F.D., Valori E., Rovellotti M.C., Done E., Faron G., Gucciardo L., Esposito V., Vena F., Giancotti A., Brunelli R., Muzii L., Nappi L., Sorrentino F., Vasciaveo L., Liberati M., Buca D., Leombroni M., Di Sebastiano F., Di Tizio L., Gazzolo D., Franchi M., Ianniciello Q.C., Garzon S., Petriglia G., Borrello L., Nieto-Calvache A.J., Burgos-Luna J.M., Kadji C., Carlin A., Bevilacqua E., Moucho M., Pinto P.V., Figueiredo R., Morales Roselló J., Loscalzo G., Martinez-Varea A., Diago V., Jimenez Lopez J.S., Aykanat A.Y., Cosma S., Carosso A., Benedetto C., Bermejo A., May Feuerschuette O.H., Uyaniklar O., Ocakouglu S.R., Atak Z., Gündüz R., Haberal E.T., Froessler B., Parange A., Palm P., Samardjiski I., Taccaliti C., Okuyan E., Daskalakis G., Moreira de Sa R.A., Pittaro A., Gonzalez-Duran M.L., Guisan A.C., Genç Ş.Ö., Zlatohlávková B., Piqueras A.L., Oliva D.E., Cil A.P., Api O., Antsaklis P., Ples L., Kyvernitakis I., Maul H., Malan M., Lila A., Granese R., Ercoli A., Zoccali G., Villasco A., Biglia N., Madalina C., Costa E., Daelemans C., Pintiaux A., Cueto E., Hadar E., Dollinger S., Brzezinski Sinai N.A., Huertas E., Arango P., Sanchez A., Schvartzman J.A., Cojocaru L., Turan S., Turan O., Di Dedda M.C., Molpeceres R.G., Zdjelar S., Premru-Srsen T., Cerar L.K., Druškovič M., De Robertis V., Stefanovic V., Nupponen I., Nelskylä K., Khodjaeva Z., Gorina K.A., Sukhikh G.T., Maruotti G.M., Visentin S., Cosmi E., Ferrari J., Gatti A., Luvero D., Angioli R., Puri L., Palumbo M., D'Urso G., Colaleo F., Chiara Rapisarda A.M., Carbone I.F., Mollo A., Nazzaro G., Locci M., Guida M., Di Spiezio Sardo A., Panici P.B., Berghella V., Flacco M.E., Manzoli L., Bifulco G., Scambia G., Zullo F., D'Antonio F. (2020 Nov 26). Risk factors associated with adverse fetal outcomes in pregnancies affected by Coronavirus disease 2019 (COVID-19): a secondary analysis of the WAPM study on COVID-19. J. Perinat. Med..

[27] Grgić G., Cerovac A., Hudić I., Laganà A.S., Favilli A., Garzon S., Chiantera V., Margioula-Siarkou C., Hadžimehmedović A., Mandžić A. (2022 Sep 9). Clinical manifestation and obstetric outcomes in pregnant women with SARS-CoV-2 infection at delivery: a retrospective cohort analysis. J Pers Med.

